# Usability and Feasibility Evaluation of a Web-Based and Offline Cybersecurity Resource for Health Care Organizations (The Essentials of Cybersecurity in Health Care Organizations Framework Resource): Mixed Methods Study

**DOI:** 10.2196/50968

**Published:** 2024-04-11

**Authors:** Niki O'Brien, Roberto Fernandez Crespo, Fiona O'Driscoll, Mabel Prendergast, Deeph Chana, Ara Darzi, Saira Ghafur

**Affiliations:** 1 Institute of Global Health Innovation Imperial College London London United Kingdom; 2 Institute for Security Science and Technology Imperial College London London United Kingdom

**Keywords:** acceptability, cross sectional, cybersecurity, digital health, digital transformation, education, feasibility, framework, frameworks, global health, health systems, implementation, organization, organizational, organizations, patient safety, SWOT, TAM, usability

## Abstract

**Background:**

Cybersecurity is a growing challenge for health systems worldwide as the rapid adoption of digital technologies has led to increased cyber vulnerabilities with implications for patients and health providers. It is critical to develop workforce awareness and training as part of a safety culture and continuous improvement within health care organizations. However, there are limited open-access, health care–specific resources to help organizations at different levels of maturity develop their cybersecurity practices.

**Objective:**

This study aims to assess the usability and feasibility of the Essentials of Cybersecurity in Health Care Organizations (ECHO) framework resource and evaluate the strengths, weaknesses, opportunities, and threats associated with implementing the resource at the organizational level.

**Methods:**

A mixed methods, cross-sectional study of the acceptability and usability of the ECHO framework resource was undertaken. The research model was developed based on the technology acceptance model. Members of the Imperial College Leading Health Systems Network and other health care organizations identified through the research teams’ networks were invited to participate. Study data were collected through web-based surveys 1 month and 3 months from the date the ECHO framework resource was received by the participants. Quantitative data were analyzed using R software (version 4.2.1). Descriptive statistics were calculated using the mean and 95% CIs. To determine significant differences between the distribution of answers by comparing results from the 2 survey time points, 2-tailed *t* tests were used. Qualitative data were analyzed using Microsoft Excel. Thematic analysis used deductive and inductive approaches to capture themes and concepts.

**Results:**

A total of 16 health care organizations participated in the study. The ECHO framework resource was well accepted and useful for health care organizations, improving their understanding of cybersecurity as a priority area, reducing threats, and enabling organizational planning. Although not all participants were able to implement the resource as part of information computing technology (ICT) cybersecurity activities, those who did were positive about the process of change. Learnings from the implementation process included the usefulness of the resource for raising awareness and ease of use based on familiarity with other standards, guidelines, and tools. Participants noted that several sections of the framework were difficult to operationalize due to costs or budget constraints, human resource limitations, leadership support, stakeholder engagement, and limited time.

**Conclusions:**

The research identified the acceptability and usability of the ECHO framework resource as a health-focused cybersecurity resource for health care organizations. As cybersecurity in health care organizations is everyone’s responsibility, there is potential for the framework resource to be used by staff with varied job roles. Future research needs to explore how it can be updated for ICT staff and implemented in practice and how educational materials on different aspects of the framework could be developed.

## Introduction

Cybersecurity is a growing challenge for health systems worldwide as the rapid adoption of emerging technologies in health care has led to increased vulnerabilities to cyber threats; these threats can significantly erode public trust and compromise patient safety [1]. While the challenge has been increasing for some time, the COVID-19 pandemic put the health sector into the spotlight, leading to increased numbers of cyberattacks during the pandemic period. Such was the scale of activity in this period that the World Health Organization (WHO) reported a 5-fold increase in attempted cyberattacks directed at WHO staff and the wider public and called for increased vigilance globally [2]. Health care systems need to be prepared for threats to continuous operations and develop their resilience in a world with substantial and accelerated technological changes.

The health sector has unique vulnerabilities to cyberattacks, most importantly the threat to patient safety and the potential loss of human life. Other unique vulnerabilities include limited funding available (both for information computing technology [ICT] services and cybersecurity), particularly in public institutions, resulting in a lack of human resources and the ability to address legacy infrastructure across health systems. Cybercriminals have forced health and social care providers to pay large sums in ransom following cyberattacks to regain access to vital technology and systems that are essential for the day-to-day functioning and care of patients [3,4]. Excluding ransom sums demanded by hackers, the average cybersecurity incident costs a health care organization US $10 million [5].

Research into health care cybersecurity has highlighted important vulnerabilities at the global level. While there are a range of technological challenges in securing medical devices and systems, human error is the primary driver of cyber breaches. Estimates from electronic health record breaches in the United States suggest that 73% of incidents were due to poor human security (eg, carelessness or negligence and falling victim to phishing scams) [6]. In May 2021, the Irish health care system was struck by the Conti ransomware attack through a phishing email with a malicious Microsoft Excel (Microsoft Corporation) file attached, which affected more than 80% of its ICT infrastructure [7]. The implications of the attack on patients and services were substantial, as staff were locked out of systems and patient care was disrupted for several months [8]. Despite these challenges, there remains a lack of commonality of language and published global documents from multilateral organizations that provide comprehensive guidance for the health sector in strengthening cybersecurity.

Given the high stakes of maintaining secure health care ICT systems, it is critical to develop workforce awareness and training as part of a safety culture and continuous improvement within health care organizations. While there is limited research, current evidence suggests that providing cybersecurity training to staff is associated with improved cyber hygiene practices, including reduced phishing email click rates [9]. Although most cybersecurity toolkits and frameworks focus on practices across all critical sectors and are not specific to the health care context, more guidance for the health sector has been developed in recent years, including the Health Care and Public Health Sector Cybersecurity Framework Implementation Guide [10]. Nonetheless, such guidance is in its infancy, and there remain limited open-access, health care–specific resources to help organizations at different levels of maturity develop their cybersecurity practices.

**Figure 1:**
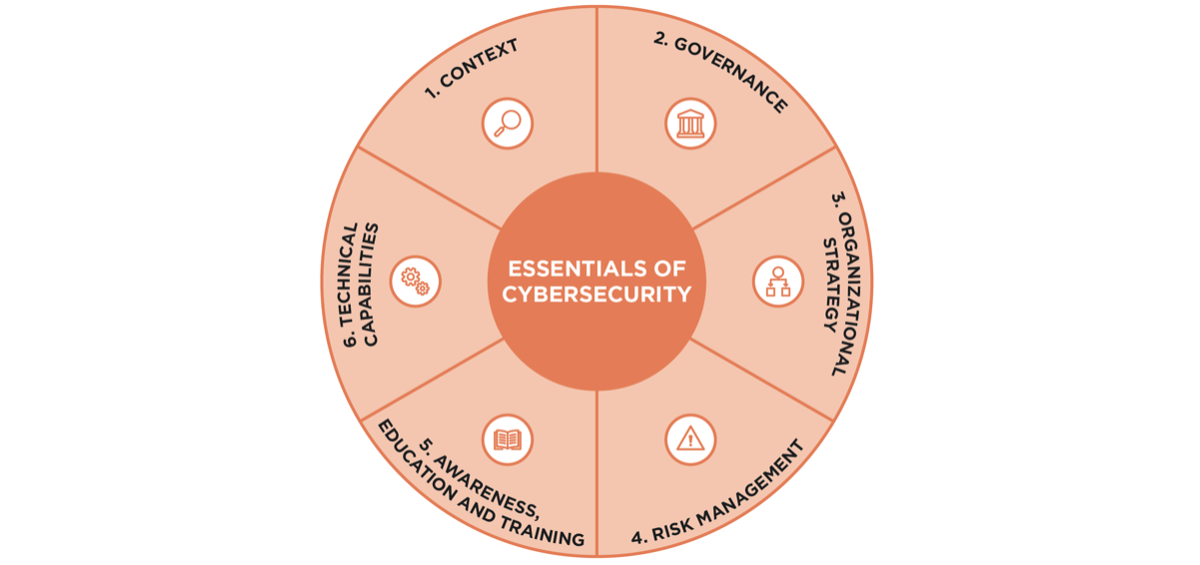
Visual representation of the 6 components that make up the Essentials of Cybersecurity in Health Care Organizations (ECHO) framework.

In 2020, the Institute of Global Health Innovation, Imperial College London, published the Essentials of Cybersecurity in Health Care Organizations (ECHO) framework, developed through research on capacity and maturity levels across health care organizations worldwide and Delphi research with leading figures in the fields of cybersecurity, ICT, and health policy [11,12]. The ECHO framework, which includes 6 dimensions (Figure 1 [11]), outlines the most important elements for health care organizations to consider and can act as a “minimum standard” or an aspirational checklist, depending on an organization’s resources and its cyber maturity. Following its release, the research team worked with cybersecurity experts and web developers to create the web-based ECHO framework resource. This resource provides key guidance based on each component of the ECHO framework and a checklist that can be used by health care organizations to track progress. As the ECHO framework resource and checklist is designed to be used across high-, middle-, and low-income countries with varied critical infrastructure, it can also be downloaded and used offline.

This study aimed to assess the usability and feasibility of the ECHO framework resource. A secondary aim was to evaluate the strengths, weaknesses, opportunities, and threats associated with implementing the ECHO framework resource at the organizational level.

## Methods

### Study Design

We conducted a mixed methods, cross-sectional study of the acceptability and usability of the ECHO framework at individual health care organizations. A convergent parallel (also known as simultaneous triangulation) mixed methods design was used, which involved conducting qualitative and quantitative components simultaneously and giving them equal priority. The research team kept both components independent during data collection and analysis and only mixed data during interpretation. This design enabled the collection of different but complementary data on the same topic, which offers the benefits of reducing the limitations of qualitative or quantitative methodologies on their own, triangulating findings, and developing a fuller understanding of the research subject [13].

### Ethical Considerations

The study protocol was approved by the Imperial College’s Science, Engineering, and Technology Research Ethics Committee (21IC6775). All participants were emailed information about the study, including a full participant information sheet, and asked if they would be interested in taking part. Participants were made aware that their participation is voluntary, and they were free to withdraw at any stage of the study by contacting the research team. Signed participant consent was received from all participants. The data collected were stored securely on the Imperial College London OneDrive and accessed on Imperial-owned, password-protected computers. Survey responses were pseudonymized, with geographic coordinates, IP addresses, names, and contact details removed before the data analysis, and stored in secure folders only accessible to the research team. Participants in the research were not compensated.

### Model

The research model was developed based on the technology acceptance model (TAM) [14]. The TAM measurement scale assesses an individual’s acceptance of technology based on perceived usefulness and perceived ease of use, which are hypothesized to be fundamental determinants of user acceptance. Though not developed for the health sector, the TAM has been used by health researchers across disciplines to measure the acceptance of digital technologies in health care and education settings [15-17].

Survey questions were adapted from previously published scales. The survey instrument was tested for clarity and comprehensiveness with 1 methodological expert and 1 ICT professional before implementation. We included additional elements of the technology acceptance model-2 (TAM2) framework, specifically subjective norm (eg, perception of the organizational factors and individuals’ approval or disapproval), job relevance (eg, the extent of relevance of the resource to job function), and attitude (eg, extent of positive perception of the resource topic). Using a 5-point Likert scale for each construct, the quantitative survey (Multimedia Appendix 1) assessed (1) perceived usefulness, (2) perceived ease of use, (3) attitude, (4) intention to use, (5) job relevance, and (6) organizational factors or external control.

### Material

**Figure 2:**
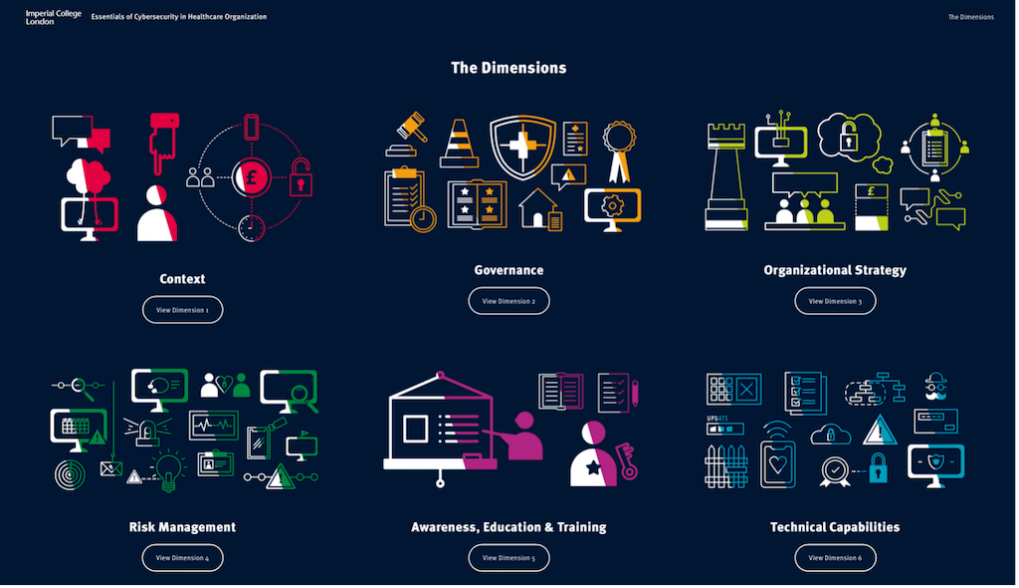
Essentials of Cybersecurity in Health Care Organizations (ECHO) framework resource website content (home page).

Participants were provided with a URL, website link, and access password to the web-based ECHO framework resource and written instructions on how to navigate the content and checklist. Participants were also informed how they could download the content and checklist for offline use. When designing the web-based resource, care was taken to ensure the website was easy to navigate and clearly identified the 6 core dimensions of the ECHO framework (Figure 2). The website was hosted on Squarespace, with website traffic encrypted by Secure Sockets Layer (SSL).

### Participants and Recruitment

Members of the Imperial College Leading Health Systems Network and other health care organizations identified through the research teams’ networks were invited to participate in the research by email. The invitation email outlined the requirement for an individual with ICT or cybersecurity oversight within the organization to participate on the organization’s behalf. Convenience sampling was used, and participants from mixed ICT roles, depending on the organizational structure, were included. The inclusion criteria were health care provider organization that uses an individual or individuals with an ICT- or cybersecurity-focused job role. Participants were excluded if they were not health care providers or did not have an ICT or cybersecurity function.

### Data Collection and Analysis

Study data were collected through Qualtrics web-based surveys 1 month and 3 months from the date the ECHO framework resource was received by the participants. The survey instrument (Multimedia Appendix 1) was broken down into 2 parts. Part 1 explored technology acceptance quantitatively (based on the TAM or TAM2 framework). Part 2 explored technical and content acceptability, feasibility, and usability qualitatively (including strengths, weaknesses, opportunities, and threats analysis).

### Quantitative Analysis

Quantitative data were analyzed using R software (version 4.2.1; R Foundation for Statistical Computing). Descriptive statistics of the answers for each survey question were calculated by calculating the mean and 95% CIs. To determine significant differences between the distribution of answers by comparing results from the 2 survey time points, 2-tailed *t* tests were used. The significance level for all statistical tests was set at a *P* value of <.05, and a 2-sided hypothesis was considered for all tests. Analysis was conducted by comparing all responses from surveys across time points as well as by comparing survey respondents based on whether they were public or private organizations and whether they were from high-income countries (HICs) or low- and middle-income countries (LMICs).

### Qualitative Analysis

Following completion of the survey data collection on Qualtrics, we imported and qualitatively compared the long-form survey responses from the baseline, 1-month, and 3-month surveys through directed content analysis in Microsoft Excel.

The thematic analysis relied largely on the use of deductive coding, which uses a top-down approach to making connections and categorizing themes under the TAM framework. As such, the 4 nodes that formed the starting point of the analysis were the process of change, acceptability and feasibility, content appraisal, and experiences with the framework. The research team also used an inductive approach to capture additional concepts, using a line-by-line review of long-form responses to derive additional codes with regard to experiences of implementing the framework. The 2 researchers independently coded the long-form responses from the baseline (NO and FO) and 1-month and 3-month surveys (NO and MP). A third research team member (SG) was used to ensure that codes were clearly defined and being applied consistently.

## Results

### Participant Characteristics

In total, 16 health care organizations participated in the baseline survey. A total of 14 (87%) health care organizations went on to complete the 1-month survey and 12 (75%) completed the 3-month survey. Table 1 outlines the sector and country each participant organization belonged. Participants represented a variety of country classifications as defined by the World Bank [18], with baseline participation from low-income countries (LICs; n=3, 18%), LMICs (n=4, 25%), middle-income countries (n=3, 18%), and HICs (n=6, 37%). Different types of health care organizations were also represented from across the public (n=9, 56%), private (n=6, 37%), and nongovernmental organization (NGO; n=1, 6%) sectors.

**Table 1:** Study participant population characteristics.

Characteristics			Baseline (N=16), n (%)		1 month (n=14), n (%)		3 months (n=12), n (%)
**Country**							
	Canada	1 (6)		1 (7)		1 (8)	
	Colombia	1 (6)		1 (7)		1 (8)	
	Ethiopia	1 (6)		1 (7)		1 (8)	
	Hong Kong	1 (6)		1 (7)		1 (8)	
	Iceland	1 (6)		1 (7)		1 (8)	
	India	1 (6)		1 (7)		1 (8)	
	Nigeria	1 (6)		1 (7)		1 (8)	
	Norway	1 (6)		1 (7)		0 (0)	
	Pakistan	3 (18)		2 (14)		1 (8)	
	Singapore	1 (6)		1 (7)		1 (8)	
	South Africa	1 (6)		1 (7)		1 (8)	
	Tanzania	1 (6)		0 (0)		0 (0)	
	Thailand	1 (6)		1 (7)		1 (8)	
	United Kingdom	1 (6)		1 (7)		1 (8)	
**Country classification**							
	LIC^a^	3 (18)		2 (14)		2 (16)	
	LMIC^b^	4 (25)		3 (21)		2 (16)	
	MIC^c^	3 (18)		3 (21)		3 (25)	
	HIC^d^	6 (37)		6 (42)		5 (41)	
**Sector**							
	Public	9 (56)		9 (64)		8 (66)	
	Private	6 (37)		4 (28)		3 (25)	
	NGO^e^	1 (6)		1 (7)		1 (8)	

^a^LIC: low-income country.

^b^LMIC: low- and middle-income country.

^c^MIC: middle-income country.

^d^HIC: high-income country.

^e^NGO: nongovernmental organization.

### Baseline Analysis

Participants in the baseline survey reported ECHO framework resource usefulness across 2 domains: framework effects and framework features. The framework effects considered useful were the ability to better improve cybersecurity in health care organizations, reduce threats, and protect data. Participants also noted the framework was useful in prompting the development of cybersecurity policies and protocols and in facilitating organizational evaluation of cybersecurity practices. The framework features participants considered most useful were its health industry focus, its comprehensiveness, its actionability, and the ease with which it could be understood.

Participants reported what they liked most across 3 domains: content, structure or presentation, and perspective taken. The content areas specifically highlighted as most liked were “Dimension 2: Governance” and “Dimension 3: Organizational Strategy.” On structure or presentation, respondents liked the clear, concise, and focused design of the ECHO framework resource. As reported when asked about usefulness, the health sector focus, as well as a focus on “the human side” of cybersecurity and that the ECHO framework was complementary to other frameworks, were most liked. Participants reported what they least liked across 2 domains: content and framework features. Several domains and components were mentioned as aspects that individual respondents did not like, along with a lack of methodology and information on how to assess cybersecurity in the resource. Some respondents also noted that there was no comparison with other frameworks within the resource. Regarding framework features, participants noted a high level and lack of detail as the aspect least liked.

**Figure 3:**
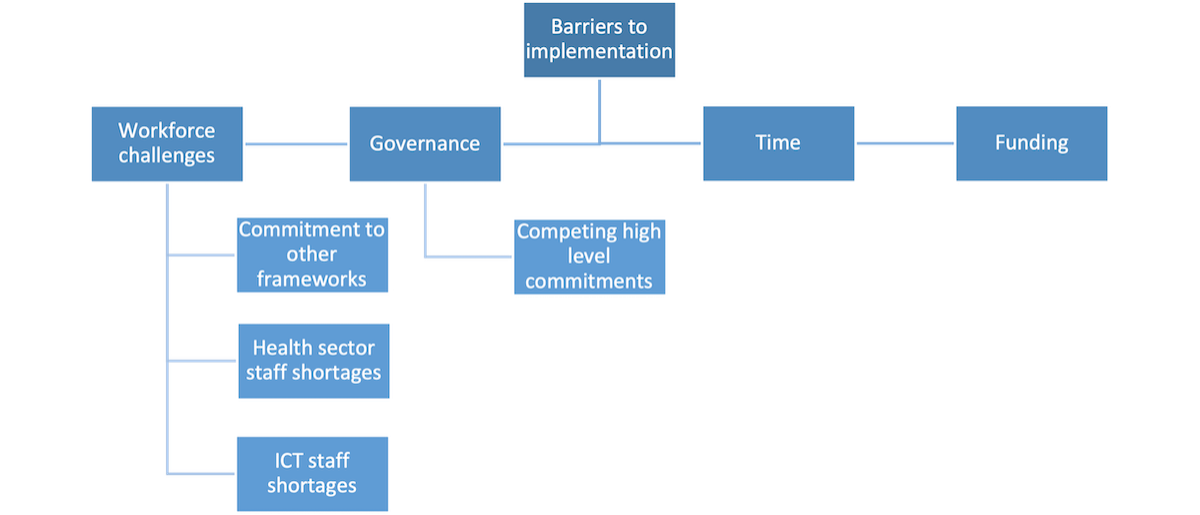
Concept map of identified barriers to implementing the framework. ICT: information computing technology.

Figure 3 outlines the barriers to implementation described by participants in the baseline survey. Workforce challenges were noted based on an existing organizational commitment to other frameworks, health sector staff shortages, and ICT staff shortages. Competing high-level commitments were outlined as a governance challenge. Time and funding to implement the ECHO framework resource were also reported as challenges by respondents.

### Quantitative Analysis Results

**Figure 4:**
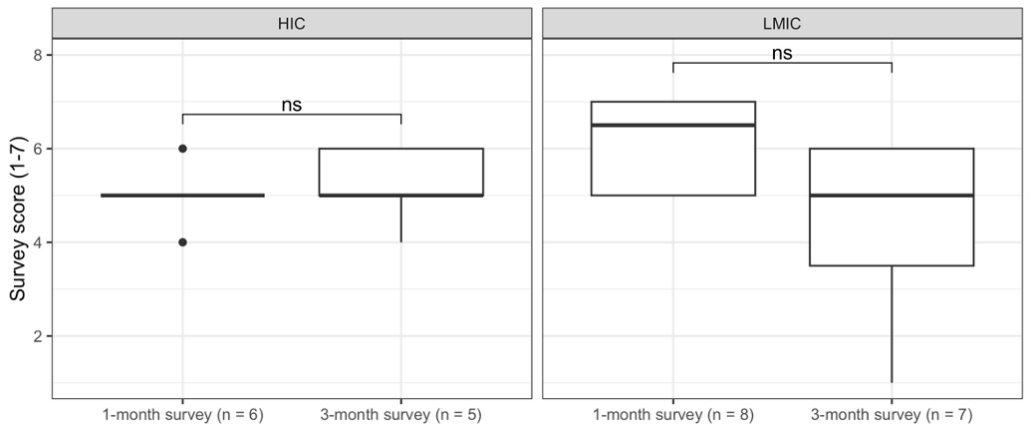
Subgroup analysis of Essentials of Cybersecurity in Health Care Organizations (ECHO) cybersecurity resource usefulness. HIC: high-income country; LMIC: low- and middle-income country and middle-income country; ns: not significant.

The TAM explores the attitudes the respondents have regarding the usefulness of the technology. Across the 2 surveys, the lowest mean score on usefulness statements (4.46, SD 1.38) was in response to “Using the ECHO framework makes it easier to do my job,” while the highest mean score (5.23, SD 1.33) was for “Using the ECHO framework for cybersecurity improves the quality of work I do.” In comparing responses to “Using the ECHO framework for cybersecurity improves the quality of the work I do” between participants in HICs and LMICs between 1- and 3-month surveys, there was a trend toward significance in the LMIC subgroup analysis over time (*P*=.06), where the mean Likert score reduced from 6.12 (SD 0.99) to 4.42 (SD 1.90; Figure 4), but this was not statistically significant.

Responses to ease-of-use statements ranged from the lowest average Likert score (5.04, SD 1.04) in response to the statement “Interacting with the ECHO cybersecurity framework is often encouraging” to the highest score (5.27, SD 1.15) to “The ECHO cybersecurity framework provides helpful guidance in performing tasks.” Mean scores for attitude questions, including “I think the ECHO framework for cybersecurity scale up is a good/wise idea” and “I am positive towards the ECHO framework for cybersecurity,” were consistently above 5.

External control questions explored the impact of external forces on the usability and acceptability of the ECHO framework resource. While mean scores related to the statement “I have no difficulty accessing and using the ECHO framework for cybersecurity in online and/or PDF format” remained above 5 across the 1- and 3-month surveys, subgroup analysis revealed a trend toward a significant increase in mean score in the 3-month survey among HIC participants (*P*=.09), but this was not statistically significant.

**Figure 5:**
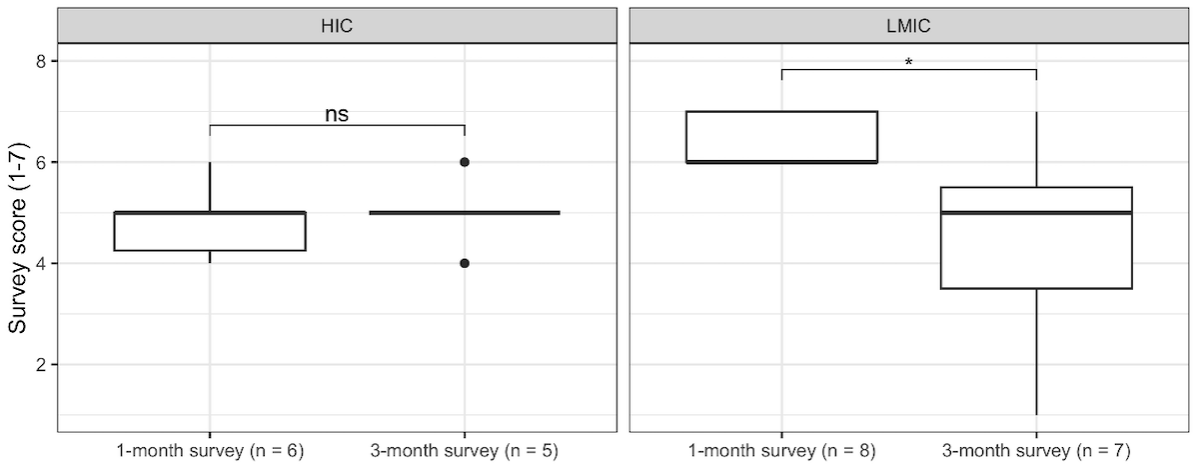
Subgroup analysis of awareness, education, and training component usefulness. ECHO: Essentials of Cybersecurity in Health Care Organizations; HIC: high-income country; LMIC: low- and middle-income country; ns: not significant.

Mean responses to how well the 6 components of the ECHO framework captured the cybersecurity needs of the organization were as follows: context (5.19, SD 1.36); governance (5.00, SD 1.13); organizational strategy (5.07, SD 1.23); risk management (5.08, SD 1.23); awareness, education, and training (5.23, SD 1.36); and technical capabilities (5.08, SD 1.32). While not significant in the HIC group, subgroup analysis of responses on awareness, education, and training showed a significant (*P*=.04) reduction in responses between the 1-month and 3-month survey among LMIC participants (Figure 5). Mean responses on ease of adoption were as follows: context (4.77, SD 1.33); governance (4.61, SD 1.27); organizational strategy (4.50, SD 1.50); risk management (4.42, SD 1.30); awareness, education, and training (5.04, SD 1.25); and technical capabilities (4.54, SD 1.36). In subgroup analysis, changes to the scoring of the adaptability of risk management in public sector facilities trended toward significant (*P*=.06), but this was not statistically significant.

### Qualitative Analysis Results

#### Acceptability and Feasibility

Thematic analysis of responses describing the experience of implementing the resource outlined varied experiences between organizations. Some respondents were unable to implement the resource, while others had only partly implemented it by the time they completed the 1-month survey. Of those who had used the resource, the experience was described as positive, based on it being easy to use and understand, and negative, based on its redundancy based on existing guidelines and tools used by some health care organizations. In responses to the 3-month survey, more participants reported that they had implemented the survey and noted that it had been useful in discussions with organizational leadership but required time to communicate it to all relevant stakeholders.

Participants most liked that the ECHO resource was well structured, user-friendly, comprehensive, current, relevant, easy to understand, teachable, practical, and globally relevant. It was also noted that the checklist was straightforward. They least liked that the resource can only be used as a reference, the time needed to implement, specific dimensions (eg, context, governance, awareness, education, and training dimensions), and the checklist functionality. The need for digital literacy was also noted as a challenge in some LMIC contexts.

#### Process of Change

Participant responses were positive in response to the process of change questions, although some noted that they were unable to implement the ECHO framework resource in their practice. Thematic analysis of 1-month survey responses to the question “What have you learned from the ECHO framework?” included the usefulness of resources for raising awareness, usefulness as a reference guide, and its ease of use based on familiarity with other standards, guidelines, and tools (eg, NIST, ISO027001, and other frameworks). A non-ECHO–specific learning was the importance of feasible and sustainable cybersecurity planning. The 3-month survey responses reflected the same themes but additionally included that the ECHO framework was useful for developing organizational strategy.

Thematic analysis of responses to the question “Have you noticed any changes in how your organization is approaching cybersecurity?” found participants were taking cybersecurity more seriously or organizations making it more of a priority, putting more importance on the “people” aspect of cybersecurity, and in some health care organizations, implementing incident management processes and other aspects suggested in the ECHO framework.

#### Content Appraisal

Participants noted the context section was difficult to engage with, particularly in relation to implementation costs, cultural factors, and staff willingness.

It was noted that developing best practice in cybersecurity, as discussed in the governance dimension, sounds straightforward but seems to be the hardest thing to do without proper step-by-step guidance. Specific organizational-level challenges related to the components of the dimension were also discussed, including the challenge of developing work-from-home policies, and clinical safety assessment hindered by lack of awareness from stakeholders.

Challenges raised in operationalizing the components outlined in “Dimension 3: Organizational Strategy” included budget constraints, engaging the board, getting other stakeholders involved, and a lack of time. Specific organizational challenges related to the components of the dimension were also discussed, including developing firewall protocols, communication strategies, etc.

Challenges related to risk management were specific organizational challenges faced by individual participants, including third-party or supply chain risk, asset identification and management, and simulation, due to human resource limitations. The lack of trained personnel, lack of time, and limited budget were challenges raised by several participants.

In responses to the question on “Dimension 5: Awareness, Education, and Training,” participants noted an organizational-level challenge in engaging a large employee base in cybersecurity that is not addressed in the ECHO framework but also recognized that it was a domain often neglected. One participant also noted the practical challenges in ensuring appropriate access to systems based on training and qualifications. Again, capacity challenges and a lack of time were highlighted as barriers to implementing components within the awareness, education, and training domains of the framework.

Challenges raised in operationalizing the technical capabilities components outlined in the ECHO framework included budget constraints, both in funding cybersecurity activities or personnel in the organization and in replacing legacy systems with new technology; lack of time; and difficulty acquiring human capital with appropriate cybersecurity knowledge and skills. Some organizations also reported a challenge around the large scale of the infrastructure that requires attention as part of cybersecurity activities and the regular patching and software updates required to maintain security.

### Experiences With the Framework

A limited number of participants expressed concerns about their ability to take part in the research. In baseline data collection, participants were asked, “Now that you know the detailed timeline for this feasibility study, do you think you/your organization will face difficulties in implementing the framework?” A total of 9 (56%) out of 16 participants responded “yes,” with the majority listing time and staffing resources as potential barriers to implementation. The subsequent surveys asked, “Have you had any difficulties to taking part in the study? If the answer is ‘Yes,’ what are they?” A total of 4 (29%) out of 14 participants responded “yes” in the 1-month survey, and 3 (25%) out of 12 participants responded affirmatively in the 3-month survey. Affirmative responses to the questions in the 1- and 3-month surveys came exclusively from participants in HICs, and the reasons for the difficulties in taking part were primary time barriers, followed by a lack of human and financial resources and a lack of prioritization of the study in the organization.

## Discussion

### Principle Findings

The ECHO framework resource was well accepted and useful for health care organizations, improving understanding of cybersecurity as a priority area in health care organizations, reducing threats, and enabling users to develop organizational planning. Participants particularly liked the ECHO framework’s health sector focus and the resource’s easiness to understand, comprehensiveness, and actionability. The mean score participants gave in the 1- to 3-month surveys to “Using the ECHO framework for cybersecurity improves the quality of the work I do” trended toward significant among LMIC participants, but these results were not statistically significant. This suggests a potential challenge in the long-term usefulness of the ECHO resource in its current format. Based on reported barriers to implementation, it is possible that continued engagement with the framework resource over time further challenges the constraints identified by diverting time and resources, which could be perceived to reduce its potential to improve the quality of work.

Although not all participants were able to implement the ECHO framework resource as part of ICT cybersecurity activities (see challenges noted in Figure 3), those who were able to implement were positive about the process of change. Learnings from the implementation process included the usefulness of the resource for raising awareness as a reference guide and that it was easy to use based on familiarity with other standards, guidelines, and tools. More broadly, it was reported that the resource was useful in driving discussions on the importance of cybersecurity with leadership. Some participants also noted that the introduction of the framework resource encouraged organizations to take cybersecurity more seriously, prioritize it, and put more importance on the “people” aspect of cybersecurity. Subgroup analysis of the statement “I have no difficulty accessing and using the ECHO framework for cybersecurity in online and/or PDF format” also revealed a trend toward a significant increase in mean score over time among HIC participants, suggesting the resource also became easier to use over time among this cohort, but this was not statistically significant.

Participants had overarching comments on the content appraisal of the 6 dimensions of the ECHO framework. They noted that several of the sections were difficult to operationalize due to costs or budget constraints, human resource limitations, leadership support, stakeholder engagement, and limited time. Select dimension-specific challenges noted were the challenge of operationalizing governance dimension components without step-by-step guidance and the difficulty undertaking activities to secure the large scale of the infrastructure outlined in the technical capabilities dimension in some health care organizations. “Dimension 5: Awareness, Education, and Training” received the highest mean score on how well the 6 components of the ECHO framework captured the cybersecurity needs of the organization. While recognizing this domain is often neglected, the qualitative analysis identified challenges moving forward, including how to engage a large employee base in cybersecurity. It may be for this reason that a significant reduction in mean score among LMIC participants between the 1-month and 3-month surveys was identified through subgroup analysis (Figure 5).

### Comparison With Previous Literature

Existing research notes the inadequacy of informatics and cybersecurity education among health care professionals [19,20]. Kamerer and McDermott [19] note that existing education calls for nurses to meet a minimal competency in informatics but does not outline the intersection between security with informatics and patient safety. However, findings from this study suggest nuances in the need for education and training within resource-limited environments with competing priorities. While participants frequently expressed their views on the importance of the awareness, education, and training component of the ECHO framework resource, they also noted major institutional capacity challenges. In quantitative analysis, despite this component scoring the highest on usefulness, participants significantly reduced usefulness scores over time. Taken together, these results highlight the need for increased understanding and research on balancing the need for cybersecurity education and training as a key priority in health care organizations with long-term priorities and capacity. Alami et al [21] note that appropriate cybersecurity can become a value-creation mechanism, suggesting cost-effectiveness and cost-benefit analyses of the resource may generate evidence on its longitudinal impact on financial expenditure and human resource time.

Barriers to implementing the ECHO framework resource described by participants, specifically limited time and funding, have also been echoed as implementation challenges in previous research. Branley-Bell et al [22] noted time pressure and fatigue as a barrier to secure behavior in the health care context, and financial barriers were cited in interviews with cybersecurity experts in Canadian and American health care organizations [23]. Our research findings expand on these more commonly reported barriers, as participants further noted workforce shortages and competing governance priorities, including the mandated use of other frameworks and overlapping compliance and reporting requirements, as barriers to implementing ECHO. Standardizing requirements and using select international standards would help eliminate the high burden of dealing with the many frameworks, regulations, and standards surrounding cybersecurity. This would also be consistent with previous digital health research that has found LMIC settings often have poor physical infrastructure and limited human resource capacity and expertise [24,25]. Further research is required to analyze these barriers in greater detail, including an exploration of where governance may be simplified and where additional human resources would be best placed to enable increased interaction with cybersecurity initiatives.

### Strengths and Limitations

The research study provides a comprehensive usability and feasibility analysis of a resource for developing cyber resilience within health care organizations. Developing usable resources on this topic is of particular importance as health care organizations face increasing cyber vulnerabilities as the use of digital technology in health increases and the technology itself becomes more complex. The worldwide diversity of participant organizations in the study is a key strength. Participants were from HICs, middle-income countries, and LICs and represented public, private, and NGO providers to capture diverse perspectives and a wide range of implementation experiences. Finally, the mixed methods approach used by a multidisciplinary research team leads to more nuanced insights into the process of change, acceptability and feasibility, and resource content appraisal.

The limitations of the research must also be discussed. Although there is precedent for using small sample sizes in usability research [26], the small sample size of the study challenged the ability to determine statistical significance in subgroup analyses. Another limitation is the implementation of the framework resource within ICT teams in health care organizations. While the approach enabled homogeneity among participants, the findings are not generalizable if used by other actors within health care organizations, for example, leadership or clinically facing staff. Finally, participant organizations in the research were self-selecting, with implications for the interpretation of findings, as those with more experience and interest in health care cybersecurity may have been more likely to opt into the study and influence the findings.

### Implications for Research, Practice, and Policy

Future research should build on the results of this formative research study to improve the design and content of the ECHO cybersecurity framework resource for use in health care organizations. High mean scores and positive qualitative comments by participants highlight that the resource is useful and applicable in health care organizations worldwide. However, lower mean scores on statements such as “Using the ECHO framework makes it easier to do my job,” coupled with the workforce shortages, competing governance priorities, limited time, and funding described by participants, suggest that further iteration of the resource is required to make it more responsive to these challenges. There is the potential for a larger-scale trial of version 2.0 to both gain participation from a larger group of participants internationally and validate the resource across HIC and LMIC contexts. However, version 2.0 may benefit from additional qualitative work to determine how ECHO could be made, at least in part, context-specific for different organizations and environments based on the ability to resource for implementation or adoption.

Previous research and academic commentary have highlighted the need for increased cybersecurity education and training [7,27]. As such, a key area of need for further investigation is how the ECHO framework resource can be used as a training tool on cybersecurity for all health care staff or organizational leadership, as well as an informational resource for ICT teams. For example, it may be possible to develop short educational snapshots on aspects of the framework that can be presented to all staff members in health care organizations through email updates; posters; other quick, attention-grabbing, and time-mindful actions; or short training courses that cover the basics of health care cybersecurity as presented in the ECHO framework. Care must be taken to ensure training courses enable staff to engage in cybersecurity topics and risk reduction from the beginning of their employment with the organization and through continuous learning over time while remaining mindful of the workloads and time required to engage in the learning. Such initiatives will require cocreation with intended users and additional testing on acceptability and feasibility.

Beyond the ECHO framework, the findings of the research study have further shown the perceived importance of cybersecurity standards, guidance, and tools for health care organizations. While there are several internationally recognized standards, guidance, and tools, their lack of focus on the health sector specifically neglects the unique aspect of cybersecurity as a critical element of patient safety and its implications for health care organizations in reducing avoidable harm. Existing standards, guidance, and tools are also often complex and technical language–heavy, which impacts their usability in low-resource health settings, often within LMIC health systems, where the level of knowledge of cybersecurity may be less advanced. As such, there is an urgent need for policy makers to fill the gap in providing targeted resources, such as standards to follow, to enable health care organizations to comply with international best practices. Policy makers must also enable an improved understanding of the unique challenges faced by health care organizations in developing more secure systems and how cyber resilience in this critical sector must feature as a priority as part of a national security agenda.

## Appendix

Multimedia Appendix 1 Survey instruments.

## Abbreviations

ECHO: Essentials of Cybersecurity for Health Care Organizations
HIC: high-income country
ICT: information computing technology
LIC: low-income country
LMIC: low- and middle-income country
NGO: nongovernmental organization
SSL: Secure Sockets Layer
TAM: technology acceptance model
TAM2: technology acceptance model-2
WHO: World Health Organization

## References

[1] Ghafur S, Fontana G, Martin G, Grass E, Goodman J, Darzi A (2019). Improving cyber security in the NHS. Institute of Global Health Innovation, Imperial College London.

[2] (2020). WHO reports fivefold increase in cyber attacks, urges vigilance. WHO.

[3] Winder D (2020). The University of California pays $1 million ransom following cyber attack. Forbes.

[4] (2021). Irish cyber-attack: hackers bail out Irish health service for free. BBC.

[5] IBM (2022). Cost of a data breach report 2022. IBM Security.

[6] Yeo LH, Banfield J (2022). Human factors in electronic health records cybersecurity breach: an exploratory analysis. Perspect Health Inf Manag.

[7] O'Brien N, Ghafur S, Sivaramakrishnan A, Durkin M (2022). Cyber-attacks are a permanent and substantial threat to health systems: education must reflect that. Digit Health.

[8] (2021). Conti cyber attack on the HSE: independent post incident review. PwC.

[9] Gordon WJ, Wright A, Aiyagari R, Corbo L, Glynn RJ, Kadakia J, Kufahl J, Mazzone C, Noga J, Parkulo M, Sanford B, Scheib P, Landman AB (2019). Assessment of employee susceptibility to phishing attacks at US health care institutions. JAMA Netw Open.

[10] (2023). Health care and public health sector cybersecurity framework implementation guidee. US Department of Health & Human Services.

[11] O'Brien N, Grass E, Martin G, Durkin M, Darzi A, Ghafur S (2020). Developing a globally applicable cybersecurity framework for healthcare: a delphi consensus study. BMJ Innov.

[12] O'Brien N, Martin G, Grass E, Durkin M, Ghafur S (2020). Safeguarding our healthcare systems: a global framework for cybersecurity. Forum Reports.

[13] Hadi MA, Alldred DP, Closs SJ, Briggs M (2013). Mixed-methods research in pharmacy practice: basics and beyond (part 1). Int J Pharm Pract.

[14] Davis FD (1989). Perceived usefulness, perceived ease of use, and user acceptance of information technology. MIS Quarterly.

[15] Park SY (2009). An analysis of the technology acceptance model in understanding university students' behavioral intention to use e-Learning. J Educ Techno Soc.

[16] Nguyen M, Fujioka J, Wentlandt K, Onabajo N, Wong I, Bhatia RS, Bhattacharyya O, Stamenova V (2020). Using the technology acceptance model to explore health provider and administrator perceptions of the usefulness and ease of using technology in palliative care. BMC Palliat Care.

[17] Portz JD, Bayliss EA, Bull S, Boxer RS, Bekelman DB, Gleason K, Czaja S (2019). Using the technology acceptance model to explore user experience, intent to use, and use behavior of a patient portal among older adults with multiple chronic conditions: descriptive qualitative study. J Med Internet Res.

[18] (2023). World bank country and lending groups. World Bank.

[19] Kamerer JL, McDermott D (2020). Cybersecurity: nurses on the front line of prevention and education. J Nurs Regul.

[20] Offner KL, Sitnikova E, Joiner K, MacIntyre CR (2020). Towards understanding cybersecurity capability in Australian healthcare organisations: a systematic review of recent trends, threats and mitigation. Intell Natl Secur.

[21] Alami H, Gagnon MP, Ahmed MAA, Fortin JP (2019). Digital health: cybersecurity is a value creation lever, not only a source of expenditure. Health Policy Technol.

[22] Branley-Bell D, Coventry L, Sillence E (2021). Promoting Cybersecurity Culture Change in Healthcare. https://dl.acm.org/doi/proceedings/10.1145/3453892.

[23] Wilner AS, Luce H, Ouellet E, Williams O, Costa N (2022). From public health to cyber hygiene: cybersecurity and Canada's healthcare sector. Int J.

[24] Akhlaq A, McKinstry B, Muhammad KB, Sheikh A (2016). Barriers and facilitators to health information exchange in low- and middle-income country settings: a systematic review. Health Policy Plan.

[25] Odekunle FF, Odekunle RO, Shankar S (2017). Why sub-saharan Africa lags in electronic health record adoption and possible strategies to increase its adoption in this region. Int J Health Sci (Qassim).

[26] Faulkner L (2003). Beyond the five-user assumption: benefits of increased sample sizes in usability testing. Behav Res Methods Instrum Comput.

[27] O'Brien N, Ghafur S, Durkin M (2021). Cybersecurity in health is an urgent patient safety concern: we can learn from existing patient safety improvement strategies to address it. J Patient Saf Risk Manag.

