# Supplementary material for: Usability and Feasibility Evaluation of a Web-Based and Offline Cybersecurity Resource for Health Care Organizations (The Essentials of Cybersecurity in Health Care Organizations Framework Resource): Mixed Methods Study
Source: JMIR Form Res. 2024 Apr 11;8:e50968. doi: 10.2196/50968 (PMC11046383; doi:10.2196/50968)
Supplement: Multimedia Appendix 1 [file formative_v8i1e50968_app1.docx]

**Multimedia Appendix 1**

**Data Collection 1 / Baseline collection**

**PART I: Implementing the ECHO framework**

The aim of this part of the survey is to learn more about your thoughts on the usefulness, feasibility and acceptability of implementing a framework to guide cybersecurity scale up within your organisation.

**Acceptability and feasibility**

**1. Feasibility -** On first review of the ECHO framework do you have all of the information you need to implement the framework within your organisation. If no, what is missing?

**2.** **Usefulness** - On first review of the ECHO framework do you think it will be useful for your organisation? If yes, how? If not, why not?

**3. Acceptability** - On first review of the ECHO framework, do you like the concept/idea of the framework? If yes, what do you like most about the framework?

**4. Suggestions for further improvement** - On first review of the ECHO framework, what are the aspects you like least about the framework? What aspects do you think will be least useful to your organisation?

**Experiences with the framework and research**

**5. Barriers** - Now that you know the detailed timeline for this feasibility study, do you think you/your organisation will face difficulties in implementing the framework? If the answer is “Yes”, what difficulties do you think you will face?

**6. Missed feedback.** Is there anything else you would like to tell us about the ECHO framework and the research process to date? If the answer is “Yes”, what would you like to tell us?

**Data collection 2 and 3**

**Part 1 explores technology acceptance (acceptability) quantitatively (based on the TAM/TAM2 framework).**

**Part 2 explores technical and content acceptability, feasibility, and usability qualitatively**

**Part I (each question is scored on a 7-point Likert scale)**

The aim of this part of the survey is to learn more about the useability, feasibility, and acceptability of the ECHO framework to guide cybersecurity scale up within your organisation.

**Perceived usefulness (5)**

Using the ECHO framework for cybersecurity improves the **quality of the work** I do

Using the ECHO framework for cybersecurity makes me more **effective** in my job

Using the ECHO framework for cybersecurity improves **my productivity**

Using the ECHO framework for cybersecurity makes it **easier to do my job**

I find the ECHO framework for cybersecurity **useful** in my job

**Perceived ease of use (5)**

The ECHO framework for cybersecurity is **easy for me to understand**

Interacting with the ECHO framework for cybersecurity is often **encouraging**

The ECHO framework for cybersecurity provides **helpful guidance** in performing tasks

I find it **easy to find the information** I need when using the ECHO framework for cybersecurity

I find the ECHO framework for cybersecurity **easy to use.**

**Attitude (3)**

I think the ECHO framework for cybersecurity scale-up is a **good idea**

I think the ECHO framework for cybersecurity scale-up is a **wise idea**

I am **positive** toward the ECHO framework for cybersecurity

**Intention to use (2)**

Assuming no significant barriers to the use of the ECHO framework exist, I **intend to use the** ECHO framework frequently

Assuming no significant barriers to the use of the ECHO framework exist, I **intend to check my organisation’s cyber planning** against the components of the ECHO framework for cybersecurity frequently

**Job relevance (2)**

Using the ECHO framework for cybersecurity is **relevant** to cybersecurity scale-up in my healthcare organisation

Using the ECHO framework for cybersecurity is **important** to cybersecurity scale-up in my healthcare organisation

**Organisational factors / External control (2)**

I have no difficulty **accessing and using** the ECHO framework for cybersecurity in online and/or PDF format

I have no difficulty accessing and using organisational and technical resources to help me access and use the ECHO framework for cybersecurity

**Part II**

The aim of this part of the survey is to learn more about your thoughts on useability, feasibility, and acceptability of the ECHO framework to guide cybersecurity scale up within your organisation.

**Process of change**

**1.. Process of change** - In the past 4 weeks, what have you learned from the ECHO guidance and implementing ECHO?

**2. Process of change** - In the past 4 weeks have you noticed any changes in how your organisation is approaching cybersecurity? If the answer is ‘Yes’, what are they?

**3. Process of change** - In the past 4 weeks have you noticed any differences in the effectiveness of your cybersecurity as a result implementing the ECHO framework? If the answer is ‘Yes’, what are they?

**4. Implementing change** - In the past 4 weeks have you been monitoring adherence to the ECHO framework? If the answer is ‘Yes’, how often?

**Acceptability and feasibility**

**5. Acceptability and feasibility -** How would you describe your experience in implementing the ECHO framework so far?

**6. Feasibility** - In the past 4 weeks, how much time do you estimate you have spent implementing and monitoring the implementation of the ECHO framework?

**7. Feasibility** - In the past 4 weeks, how much time do you estimate your team/wider organisation has spent implementing and monitoring the implementation of the ECHO framework?

**8. Acceptability** - What do you like most about the ECHO framework? What aspect has been most useful to your organisation?

**9. Suggestions for further improvement** - What do you like least about the ECHO framework? What aspect has been least useful to your organisation?

**Content appraisal**

**10.** How well did the components in the *Context* part of the ECHO framework capture the cybersecurity needs of your organisation in this area?

[Likert scale 1-5 (Did not capture at all - Captured very well)]

**11.** To what extent were the components in the *Context* part of the ECHO framework easy to adopt?

[Likert scale 1-5 (Very difficult to adopt - Very easy to adopt)]

If relevant, please describe which components were challenging to adopt and why?

**12.** How well did the components in the *Governance* part of the ECHO framework capture the cybersecurity needs of your organisation in this area?

[Likert scale 1-7 (Did not capture - Captured very well)

**13.** To what extent were the components in the *Governance* part of the ECHO framework easy to adopt?

[Likert scale 1-5 (Very difficult to adopt - Very easy to adopt)]

If relevant, please describe which components were challenging to adopt and why?

**14**. How well did the components in the *Organizational strategy* part of the ECHO framework capture the cybersecurity needs of your organisation in this area?

[Likert scale 1-7 (Did not capture - Captured very well)

**15.** To what extent were the components in the *Organizational strategy* part of the ECHO framework easy to adopt?

[Likert scale 1-5 (Very difficult to adopt - Very easy to adopt)]

If relevant, please describe which components were challenging to adopt and why?

**16.** How well did the components in the *Risk Management* part of the ECHO framework capture the cybersecurity needs of your organisation in this area?

[Likert scale 1-7 (Did not capture - Captured very well)

**17.** To what extent where the components in the *Risk Management* part of the ECHO framework easy to adopt?

[Likert scale 1-5 (Very difficult to adopt - Very easy to adopt)]

If relevant, please describe which components were challenging to adopt and why?

**18**. How well did the components in the *Awareness, education and training* part of the ECHO framework capture the cybersecurity needs of your organisation in this area?

[Likert scale 1-7 (Did not capture - Captured very well)

**19.** To what extent were the components in the *Awareness, education and training* part of the ECHO framework easy to adopt?

[Likert scale 1-5 (Very difficult to adopt - Very easy to adopt)]

If relevant, please describe which components were challenging to adopt and why?

**20.** How well did the components in the *Technical capabilities* part of the ECHO framework capture the cybersecurity needs of your organisation in this area?

[Likert scale 1-7 (Did not capture - Captured very well)

**21**. To what extent were the components in the *Technical capabilities* part of the ECHO framework easy to adopt?

[Likert scale 1-5 (Very difficult to adopt - Very easy to adopt)]

If relevant, please describe which components were challenging to adopt and why?

**Experiences with the framework and research**

**22. Barriers** - Have you had any difficulties to taking part in the study? If the answer is “Yes”, what were they?

**23. Missed feedback.** Is there anything else you would like to tell us about your experiences with the ECHO framework and the research process to date? If the answer is “Yes”, what would you like to tell us?
